# Study on the structure optimization and anti‐hepatitis B virus activity of novel human La protein inhibitor HBSC11

**DOI:** 10.1002/jmv.25528

**Published:** 2019-07-12

**Authors:** Shuangmei Tong, Jiaqian Pan, Jing Tang

**Affiliations:** ^1^ Department of Pharmacy The Obstetrics & Gynecology Hospital of Fudan University Shanghai China

**Keywords:** antiviral agents, drug‐like optimization, hepatitis B virus

## Abstract

In our previous study, Methyl pyrazolo[1,5‐a] pyridine‐2‐carboxylate (HBSC11) was shown to combine with La protein, which conferred anti‐hepatitis B virus (HBV) effects. The purpose of this study was to optimize, synthesize, and evaluate the anti‐HBV activity of HBSC11. The methyl group of HBSC11 was substituted with hydrophobic, hydrophilic, and tricyclic groups to generate novel HBV inhibitors with desirable potency. On in vitro evaluation, several derivatives exhibited good anti‐HBV activity compared with control. In particular, compound 5a reduced the level of HBV antigen by approximately 50%, which was similar to the activity of entecavir. In a mouse model, 5a showed 98.9% inhibition rate for HBV DNA, 57.4% for HBsAg, and 46.4% for HBeAg; the corresponding rates in the control group were 90.8, 3.8, and 9.8%, respectively. In addition, prediction of binding modes and physicochemical properties showed that 5a formed hydrogen bonds with La protein and conformed well to the Lipinski's rule of five. Our results suggest that 5a is a potential new anti‐HBV drug.

## Highlights

- La protein protects HBV RNA from destruction by combining with HBV RNA and covers up the RNA cleavage site.
- HBSC11 (Methyl pyrazolo[1,5‐a] pyridine‐2‐carboxylate) is a novel La protein inhibitor which we identified as previous.
- 10 derivatives (3a‐3f, 5a‐5d) were obtained by 2 sections‐scaffold and kept the active site form leading compound HBSC11.
- Candidate compound 5a exhibited potent anti‐HBV activity with safety concentration and satisfied physicochemical properties.

## 1 INTRODUCTION

Chronic hepatitis B virus (HBV) infection is a major global public health problem with significant morbidity and mortality.^1^, ^2^, ^3^ An estimated 2 billion people have been infected with HBV worldwide till date and approximately 780 000 patients die from HBV‐related complications every year (http://www.who.int/en/news‐room/fact‐sheets/detail/hepatitis‐b.). Currently, there are two main antiviral therapies available for patients with HBV: nucleoside analogs (NAs) and interferon‐alpha (IFNα). The NAs are generally safe; however, long‐term oral treatment with NAs may lead to the emergence of drug resistance. IFNα is expensive and causes adverse effects.^4^ Therefore, development of novel anti‐HBV agents with superior efficacy and safety is a key imperative.^5^

La protein was first identified as a ribonucleic protein (RNP) targeted by autoantibodies in patients with autoimmune diseases; it is now known to be a multifunctional nucleoprotein involved in diverse aspects of RNA metabolism and translation. La protein binds to nascent RNA polymerase III transcripts, which protects them from exonucleolytic decay; in addition, it plays a role as RNA chaperone to help them correct folding.^6^ Studies have shown that combination of La protein to HBV RNA alters its conformation and covers up the RNA cleavage site, which protects the HBV RNA from destruction^7^, ^8^, ^9^, ^10^ and helps maintain the replication ability of HBV.^11^ Mutation and silencing of La protein have been used to confirm its role in HBV.^12^ Subsequently, according to the structural characteristics of La protein crystal, Methyl pyrazolo[1,5‐a] pyridine‐2‐carboxylate (HBSC11), a pyrazolo[1,5‐a] pyridine compound, was selected as inhibitor of La protein by virtual screening and showed good anti‐HBV efficacy.^13^

In this study, we aimed towards optimization, synthesis, and evaluation of the anti‐HBV activity of HBSC11 as a specific inhibitor of La protein. This study may provide a foundation for developing new non‐nucleoside agents for anti‐HBV therapy.

## 2 MATERIALS AND METHODS

### 2.1 Structural optimization of HBSC11

**Scheme 1:**
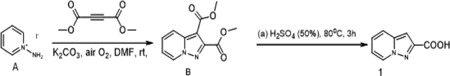
compound 1 synthetic route

**Scheme 2:**
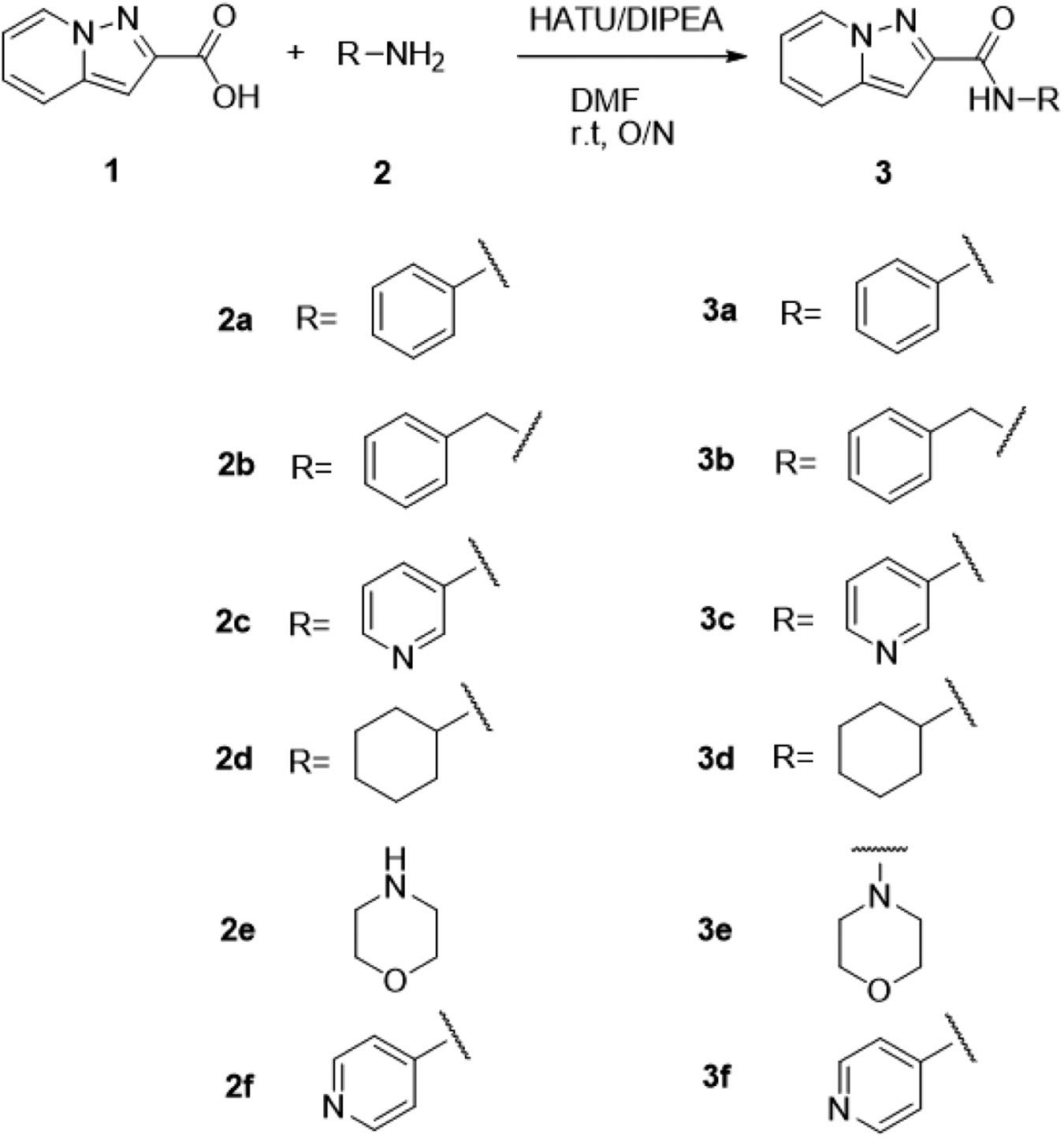
derivatives synthetic route

**Scheme 3:**
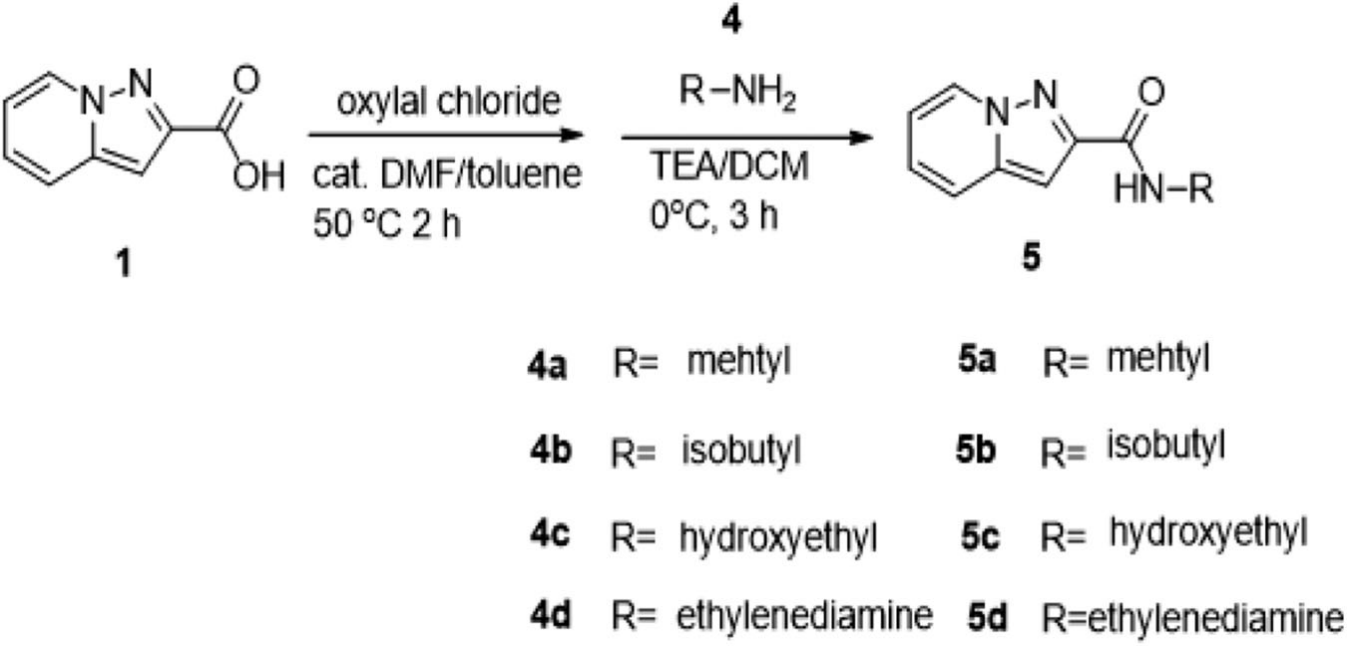
derivatives synthetic route

The derivatives of HBSC11 were designed on the basis of two sections‐scaffold: Compound 1 preparation and parallel synthesis with purification. The synthesis route of Compound 1 is outlined in Scheme 1. The synthesis route of Compounds 3a‐3f is depicted in Scheme 2, whereas that of Compounds 5a‐5d is depicted in Scheme 3. The structure of derivatives was confirmed by H Nuclear Magnetic Resonance (HNMR) and Liquid Chromatography‐Mass Spectrometry (LCMS), and the purity was analyzed by High‐Performance Liquid Chromatography (HPLC). All compounds were dissolved in Dimethyl Sulfoxide (DMSO; Sigma‐Aldrich) at 100 mM and stored at −20°C.

#### 2.1.1 Intermediate Compound 1 synthetic route

Compound A (2.22 g, 10 mmol) was suspended in dry Dimethyl Formamide (DMF) 30 mL; subsequently, dimethyl but‐2‐ynedioate (2.84 g, 20 mmol) and potassium carbonate (2.72 g, 20 mmol) were added at room temperature (RT) and stirred for 12 hours. The reaction mixture was poured into water (50 mL), then extracted with ethylacetate (EA) (100 mL × 3); the organic layer was washed with water (80 mL) and brine (80 mL), and the solvent removed under reduced pressure to afford the desired product as yellow solid. The solid was dissolved in sulfuric acid (15 mL, 50%) and then warmed to 80°C for 3 hours. The reaction mixture was poured into the water at 0°C (50 mL), and then extracted with EA (150 mL × 3); the organic layer was washed with brine (150 mL), and then dried over anhydrous Na_2_SO_4_. After filtration, the filtrate was concentrated under vacuum, the crude product was purified by thin‐layer chromatography (TLC; EA/PE = 2:1) to afford the desired Compound 1. Total yield was 46%.

#### 2.1.2 Parallel synthesis route

Compound 1 (405 mg, 2.5 mmol) and N‐Ethyldiisopropylamine (DIPEA, 967.5 mg, 7.5 mmol) were dissolved in dry DMF (5.0 mL). HATU (1‐[Bis(dimethylamino)methylene]‐1H‐1,2,3‐triazolo[4,5‐b] pyridinium 3‐oxide hexafluorophosphate, 1.43 g, 3.75 mmol) was added to the solution at 0°C, and the mixture was stirred for 10 minutes; subsequently, amine 2a~2e (1.5 eq) was added slowly and the mixture was stirred at room temperature overnight. The reaction mixture was poured into water (50 mL), and then extracted with EA (50 mL × 3); the organic layer was washed with water (50 mL) and brine (50 mL), and then dried over anhydrous Na_2_SO_4_. After filtration, the filtrate was concentrated under vacuum, and the crude product was purified by prepare TLC (PE/EA = 1:1) to afford the desired product as shown in supplement (S1).

Parallel to this, compound 1 (567 mg, 3.5 mmol) was suspended in dry toluene (20 mL), and three drops DMF and oxalyl chloride (1.0 mL, 11.8 mmol) were added at RT, then warmed to 50°C and stirred for 3 hours. The solvent was removed under reduced pressure to afford the desired product as yellow solid; the solid was dissolved in dry dichloromethane (DCM, 15 mL) and added dropwise into a solution of amine 4a~4c (3.0 eq) and Triethylamine (TEA, 1.5 mL, 10.5 mmol) in dry DCM at 0°C over 30 minutes; after addition, the mixture was further stirred for 3 hours, and the solvent removed under vacuum. The crude product was further purified by prepare TLC (PE/EA = 2:1 or pure EA) to afford the desired product as shown in supplement (S1).

### 2.2 Cell culture and biological evaluation

HepG2.2.15 and HepAD38 cells were generously provided by Xiaonan Zhang (The Research Unit, Shanghai Public Health Clinical Center, Key Laboratory of Medical Molecular Virology, School of Basic Medical Sciences of Shanghai Medical College, Fudan University), and maintained in Dulbecco's modified Eagle's medium (DMEM; Gibco) with 10% (v/v) fetal bovine serum (Gibco), penicillin 100 U/mL and streptomycin 100 μg/mL (Genom, China), and 2  mM l‐glutamine (Genom, China) at 37°C, 5% CO_2_. For experiments, 348 µg/mL G418 (Gibco) was added to the medium of HepG2.2.15 cell line and 1% doxorubicin (MCE) was used for HepAD38 cell line.

For drug treatment, HepG2.2.15 and HepAD38 cells were seeded at appropriate numbers in collagen‐coated 96‐ or 48‐well plates for different experimental purposes. HepG2.2.15 cells were cultured for 24 hours and 48 hours at 50 μM of 10 derivatives to select candidate compounds.^13^ HepAD38 cells were cultured in various concentrations of candidate compounds instead of doxorubicin for 12 days. Supernatants were harvested on day 12. The levels of HBsAg and HBeAg secreted in the culture medium were measured using ELISA Kit (Kehua, China) according to the manufacturer's protocol. Quantitative analysis of HBV DNA and HBV RNA was performed using Hepatitis B Viral DNA/RNA Quantitative Fluorescence Diagnostic Kit (ShenXiang, China) according to the manufacturer's protocol. Hirt extraction and T5 exonuclease digestion for cccDNA PCR quantification.^14^, ^15^ Cytotoxicity was assessed by CCK‐8 (Bimake) and flow cytometer.

### 2.3 Molecular modeling and molecular dynamics simulation

The three‐dimensional model of La protein was constructed using validated homology techniques and docking study using the Glide software (Glide, version 5.5, Schrödinger, LLC, New York, NY, 2009).^13^, ^16^ Binding affinity between the protein and compounds was estimated using well‐established molecular dynamics (MD) experiments.^17^, ^18^

### 2.4 Prediction of physicochemical properties

Leading compound HBSC11 and its candidate compounds 3a, 5a, and 5b were predicted for their compliance with the Lipinski's rule of five using free online software (http://www.molinspiration.com/).^19^

### 2.5 Ethical statement

The study was reviewed and approved by the Ethics Committee of the Obstetrics & Gynecology Hospital of Fudan University. All efforts were made to minimize animal suffering.

### 2.6 Hydrodynamics‐based transfection in mice

C57BL/6 mice (age: 6 weeks) were purchased from B&K Universal Group Limited (Shanghai, China). Mice were kept in pathogen‐free (SPF) environments at the animal facility. The experiments were performed during the light phase of the day. Replication‐competent HBV DNA, pAAV/HBV1.2 was a generous gift from Xiaonan Zhang. The Hydrodynamics‐based transfection mouse model was established as described by Huang et al.^20^ Ten micrograms of HBV plasmid DNA were diluted in phosphate buffered saline (PBS) in a volume equivalent to 8% of mouse body weight and then injected into the tail veins of mice within 6 to 8 seconds. Animals were killed after 12 days. Serum specimens were collected for HBV DNA, HBsAg and HBeAg assays at the indicated time‐points after injection. The livers of mice were preserved in OCT Compound (Optimum cutting temperature) for the immunohistochemical (IHC) analysis.

### 2.7 IHC staining for LA protein

The liver tissues of mice were collected from mice killed at 12 days. La protein and cell nucleus were visualized by IHC staining of tissues embedded in OCT by rabbit anti‐La (Abcam, UK) and Hoechst (Hoechst AG, Germany), respectively.

### 2.8 Statistics

The data are presented as mean ± standard deviation (n = 3). One‐way ANOVA test was performed using the SAS system (SAS Institute Inc) or GraphPad Prism version 5 (GraphPad Software Inc, San Diego) software. *P* value < .05 vs control was considered statistically significant.

## 3 RESULTS

### 3.1 Chemistry

The synthetic routes of HBSC11 derivatives 3a‐3f and 5a‐5d are illustrated in Scheme 1‐3. The key intermediate compound 1 (aminopyridine iodine) was prepared via cyclization and decarboxylic reaction with aminopyridinium iodide and dimethyl but‐2‐ynedioate. Ten derivatives of HBSC11 (Table 1) were obtained by chemical synthesis (Scheme 2 and 3) and purification. The structure of the product was confirmed by HNMR and LCMS. Results of HPLC revealed a purity of >95%.

**Table 1:** The ramification structure of compounds

No.	Molecular weight	Structure
3a	237	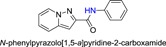
3b	251	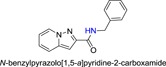
3c	238	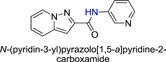
3d	243	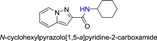
3e	231	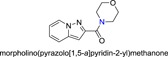
3f	238	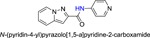
5a	175	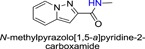
5b	217	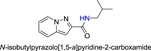
5c	205	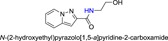
5d	204	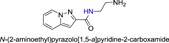

### 3.2 Biological evaluation of HBSC11 and screening for promising candidate

**Figure 1:**
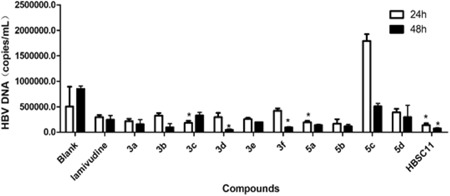
HBV DNA level in HepG2.2.15 cells after 24, 48 hours of treatment with 50 μM derivatives. HBV, hepatitis B virus

The anti‐HBV activity of HBSC11 derivatives (effect on HBV DNA replication) was assessed in HepG2.2.15 cells using lamivudine as a positive control. In the first‐round screen, HepG2.2.15 cells were treated with the test compounds at 50 μM for 24 hours and 48 hours; this was followed by quantification of the HBV DNA level by qPCR. As shown in Figure 1, some derivatives such as 3c, 3d, 3f, and 5a showed better inhibitory activity (38, 82, 62, and 34%, respectively) compared with lamivudine at the indicated time‐points. HBSC11 inhibited HBV DNA up to 54% and 71% at 24 hours and 48 hours (respectively) compared with lamivudine. Except for 5c and 5d, other derivatives showed better or equivalence with lamivudine. Considering the inhibition rate of HBV DNA at 24 hours and 48 hours, we first screened 5a, 5b, 3a, and the lead compound HBSC11 in the subsequent research.

**Figure 2:**
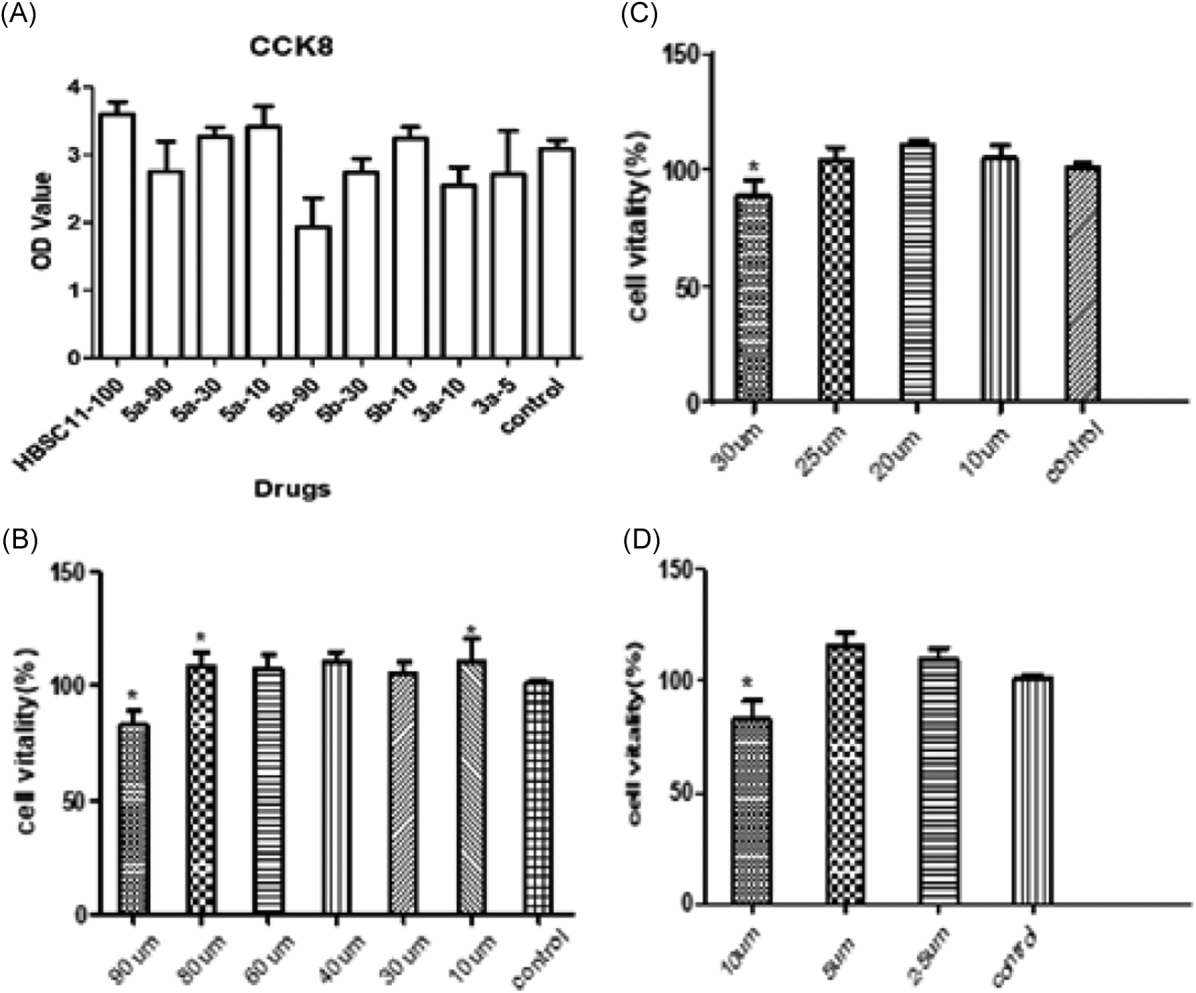
Maximum safe concentrations selected of compounds HBSC11 (A), 3a (B), 5a (C), and 5b (D). (100 µM, 80 µM, 25 µM, and 5 µM are the maximum safe concentration of compound HBSC11, 5a, 5b, and 3a, respectively. *indicates a statistically significant difference vs control group, lower or higher

**Figure 3:**
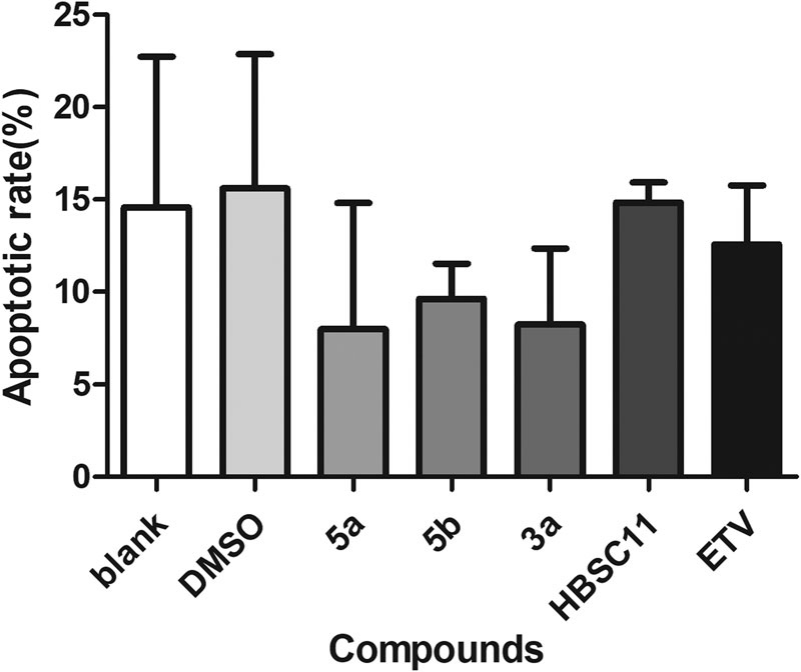
Detection of the apoptotic rate of Hep AD 38 cells after treatment with compounds. DMSO, dimethyl sulfoxide

To further confirm the anti‐HBV activity of compounds 5a, 5b, 3a, and HBSC11 in HepAD 38 cell line, the HBV DNA, cccDNA, HBV RNA, HBsAg, HBeAg, and La protein levels were evaluated after treatment with the maximum safe concentrations of compounds for 10 days. As shown in Figure 2, the maximum safe concentrations of compound HBSC11, 5a, 5b, and 3a were 100 µM, 80 µM, 25 µM, and 5 µM, respectively. All these compounds showed low cytotoxicity at the tested concentrations (Figure 3).

**Figure 4:**
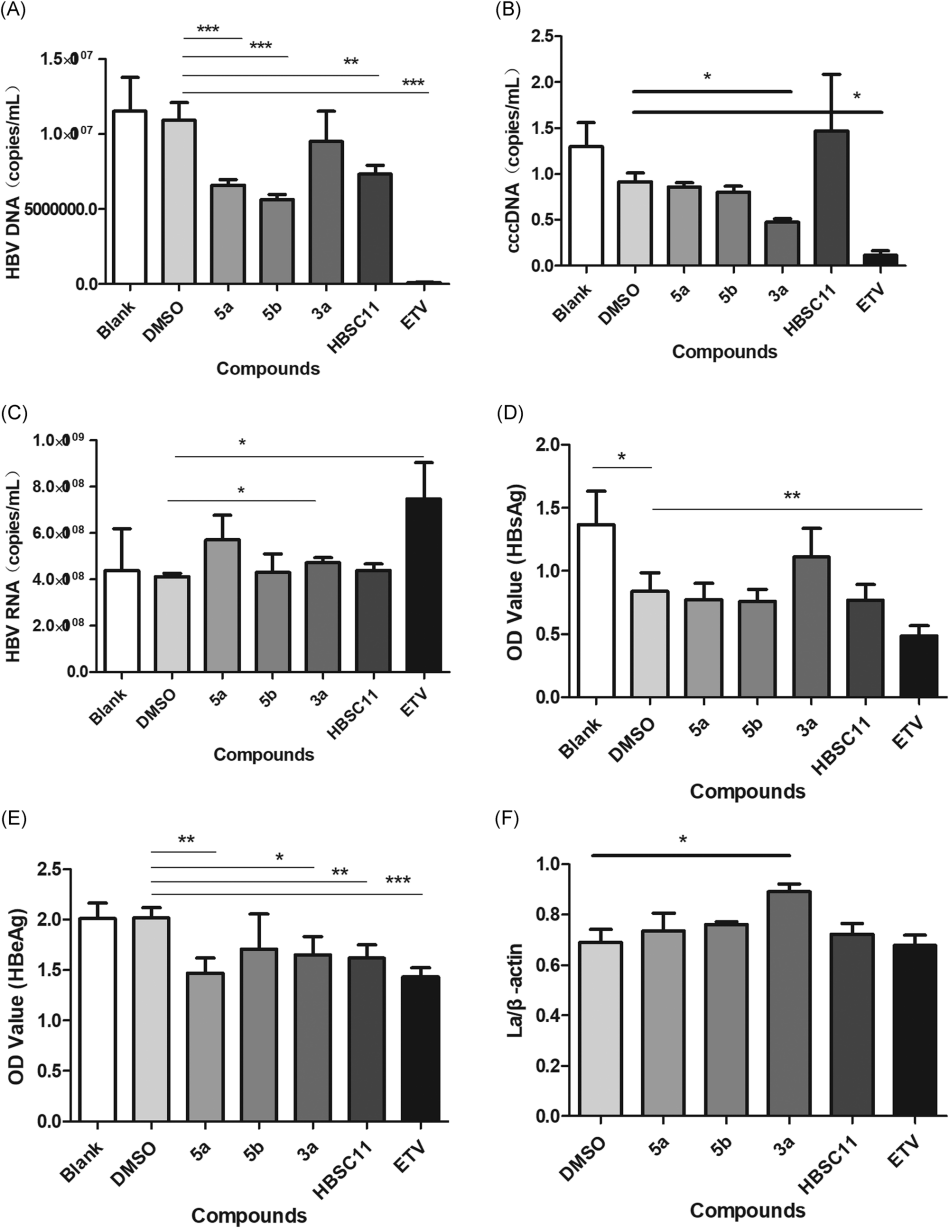
A, HBV DNA, B, cccDNA, C, HBV RNA, D, HBsAg, E, HBeAg, and F, La protein levels in HepAD 38 cells after 10 days of stimulation by candidate agents with indicated concentrations. HBV, hepatitis B virus

The extracellular HBV DNA was collected and quantified by qPCR (Figure 4A). Compounds 5b, 5a, and HBSC11 inhibited HBV DNA by up to 50%, although ETV showed better inhibitory activity. However, 3a and ETV were found preferable to 5a, 5b, and HBSC11 with respect to the effect on intracellular cccDNA (Figure 4B). On the contrary, the intracellular viral RNA level was increased upon ETV treatment (Figure 4C). Similarly, HBV DNA synthesis was significantly inhibited, whereas viral RNA was increased.^21^ The antigen inhibitory efficiency of compounds was assessed by ELISA. As shown in Figures 4D,E, compound 5a and HBSC11 showed similar effect compared with ETV in reducing the antigen levels (approximately 50%). The results indicated that the effects of our compounds on HBV were not mediated via direct inhibition of the replication of HBV DNA; however, these did show some effect on HBsAg and HBeAg. However, because the compounds did not show any effect on inhibition of La protein, we presumed that HBSC11 and its derivatives do not act directly on La protein synthesis, but on cellular localization or function (Figure 4F).

### 3.3 In vivo antiviral activity and cytotoxicity of candidate derivatives

**Figure 5:**
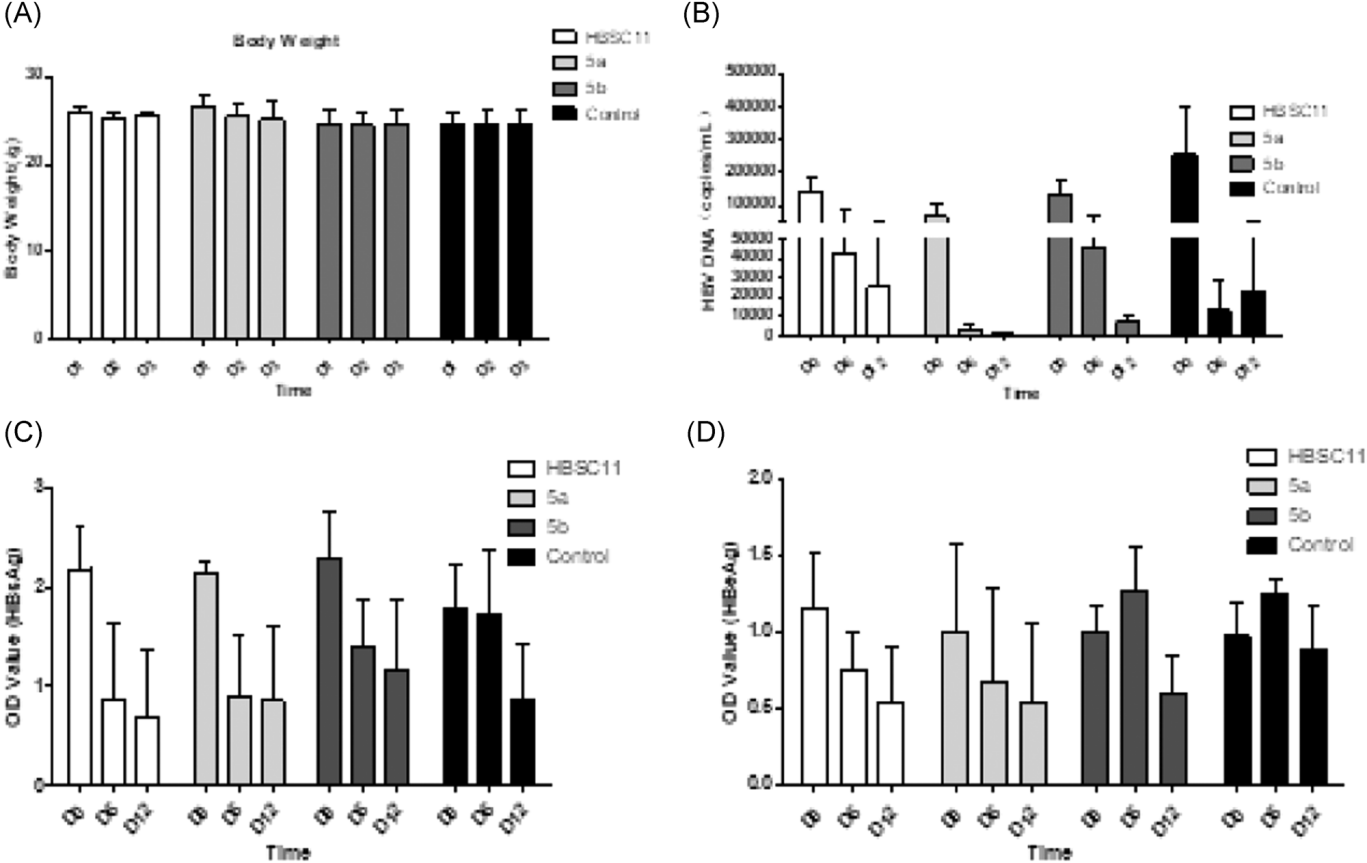
A, Body weight of mice after administration of a higher dose (500 mg/kg) for three consecutive days. HBV DNA (B), HBsAg (C), and HBeAg (D) level in mice serum after treatment with HBSC11, 5a and 5b at 0, 6, and 12 days. Compounds dissolved in the DMSO (Dimethyl sulfoxide), PEG400 (Polyethylene glycol) and ddH2O in the ratio of 1:4:5. The control was the mixture solvent. HBV, hepatitis B virus

On the basis of the results of in vitro experiments, HBSC11, 5a, and 5b were selected for assessment of antiviral activity and toxicity in vivo. The human La protein is conserved in chimpanzee, mouse, and rat among other animals (https://www.ncbi.nlm.nih.gov/gene/6741). Besides, the percent identity of human La and mouse La is 74.06%, and they share the Gln20, Lys128, Asn139, and Ile140, which can form hydrogen bonds with compounds. Therefore, we established the hydrodynamics‐based transfection mouse model to evaluate anti‐HBV activities of compounds. Because the solubility of the compound and the control volume in mice is limited to 300 microliters, we dissolved the compounds in DMSO, PEG400 (Polyethylene glycol), and ddH_2_O in the ratio of 1:4:5. The mice survived and showed no significant decrease in body weight (Figure 5A) or other signs of poisoning (depressed, decreased activity, or other symptoms) even after receiving higher dose (500 mg/kg) for three consecutive days. Considering safety, we attempted a preliminary dose of 250 mg/kg in the in vivo antiviral activity study. The results showed an anti‐HBV activity of all three compounds, which needs to be verified and further optimized (Figure 5B‐D). From the data of Figure 4, compound 5a showed the most potent in vivo anti‐HBV DNA effect with an inhibition rate of 95.9% (Day 6) and 98.9% (Day 12), although the rates of decrease were 94.7% (Day 6), and 90.8% (Day 12) in the control group. The HBsAg levels are obviously decreased to 60.1% (HBSC11) and 57.4% (5a) on day 6, whereas the control group only showed a decrease of 3.8%. Interestingly, HBSC11, 5a, and 5b reduced HBeAg up to 53.0%, 46.4%, and 40.2%, respectively, on Day 12 (only 9.8% in control group), which was consistent with the in vitro results.

**Figure 6:**
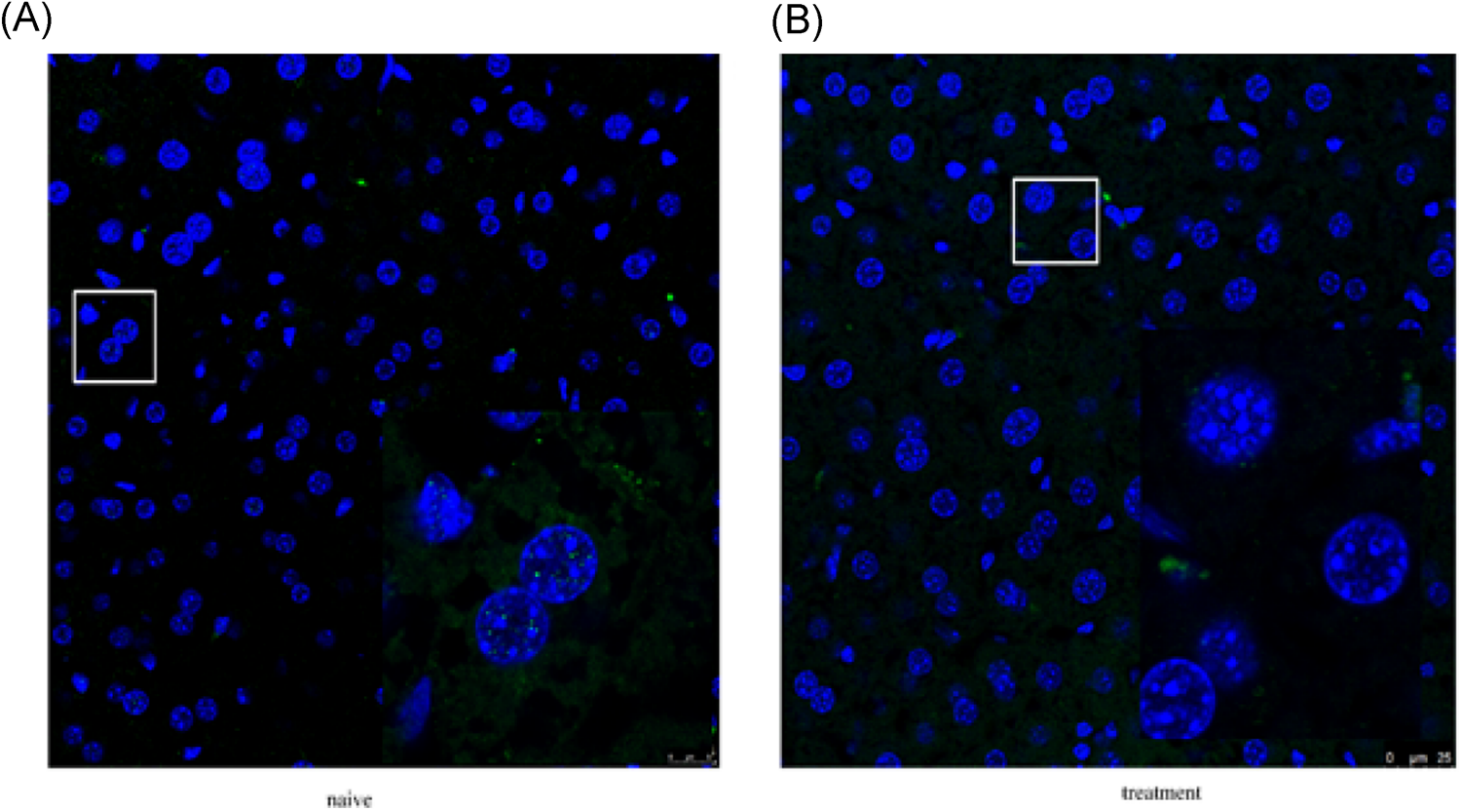
Immunohistochemical examination for La protein in hepatocytes of a control mouse (A) and treated mouse (B)

In addition, we sought to assess whether the expression of La protein in liver tissue or cell localization could be affected by the compounds. Immunofluorescence assay showed a decreasing trend of La protein in the hepatocytes after administration, especially in the nucleus; besides, there may be a tendency of protein exchange between hepatocytes and immune cells (Figure 6). Further verification of specific results and the underlying mechanisms is required in future research.

### 3.4 Molecular modeling and MD simulation study

To gain insights into the mechanism by which 3a‐3f and 5a‐5d compounds affect the biological function of La protein, we carried out docking simulation for binding modes of compounds to human La protein by Schrodinger's GLIDE components (Table 2). The results showed that compounds 3b, 3c, 3d, 5c, and 5d form two hydrogen bonds, i.e., with the oxygen atom of Ile 140 and the nitrogen atom of Asn139, respectively. Compound 5a forms two hydrogen bonds with the nitrogen atom of Ile 140 and Lys128, respectively. Compound 3e forms one hydrogen bond with the nitrogen atom of Gln20. Compound 5b forms one hydrogen bond with the oxygen atom of Ile140. Compounds 3a and 3f form one hydrogen bond with the nitrogen atom of Asn139.

**Table 2:** Predicted binding modes of compounds to the human La protein

No.	binding mode	No.	binding mode
3a	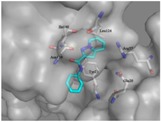	3b	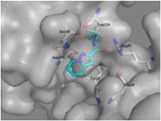
3c	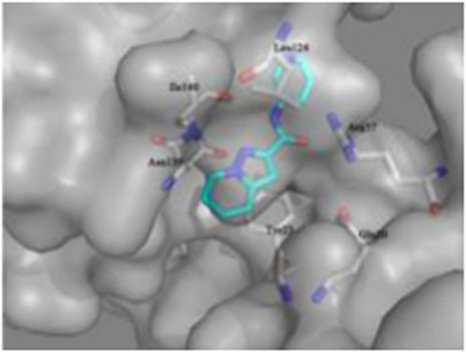	3d	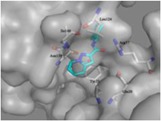
3e	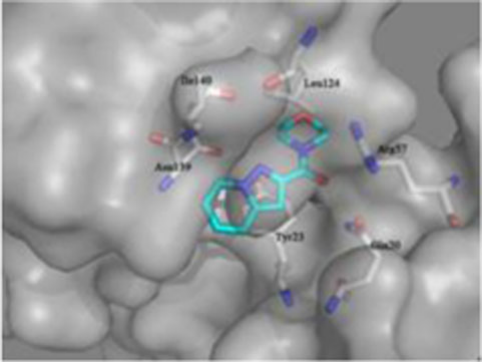	3f	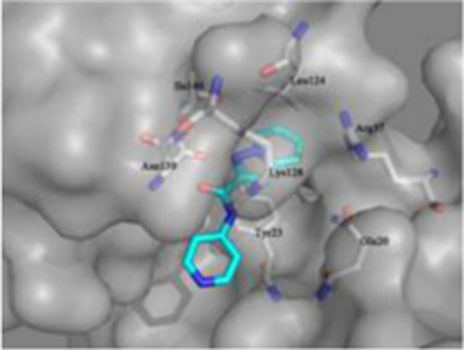
5a	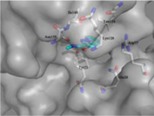	5b	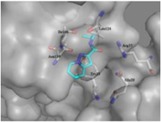
5c	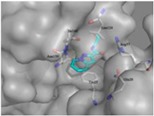	5d	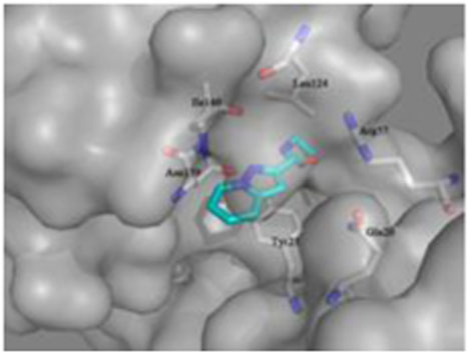

*Predicted binding modes of compounds to the human La protein. Protein surface is drawn in gray. Oxygen atoms are shown in red and nitrogen atoms in blue. Carbon atoms of compounds and protein are shown in cyan and gray, respectively. Crucial residues in the binding site are shown as a stick and labeled. Hydrogen bonds between compounds and protein are depicted by the yellow dotted line. Figures were generated by PyMol.

**Figure 7:**
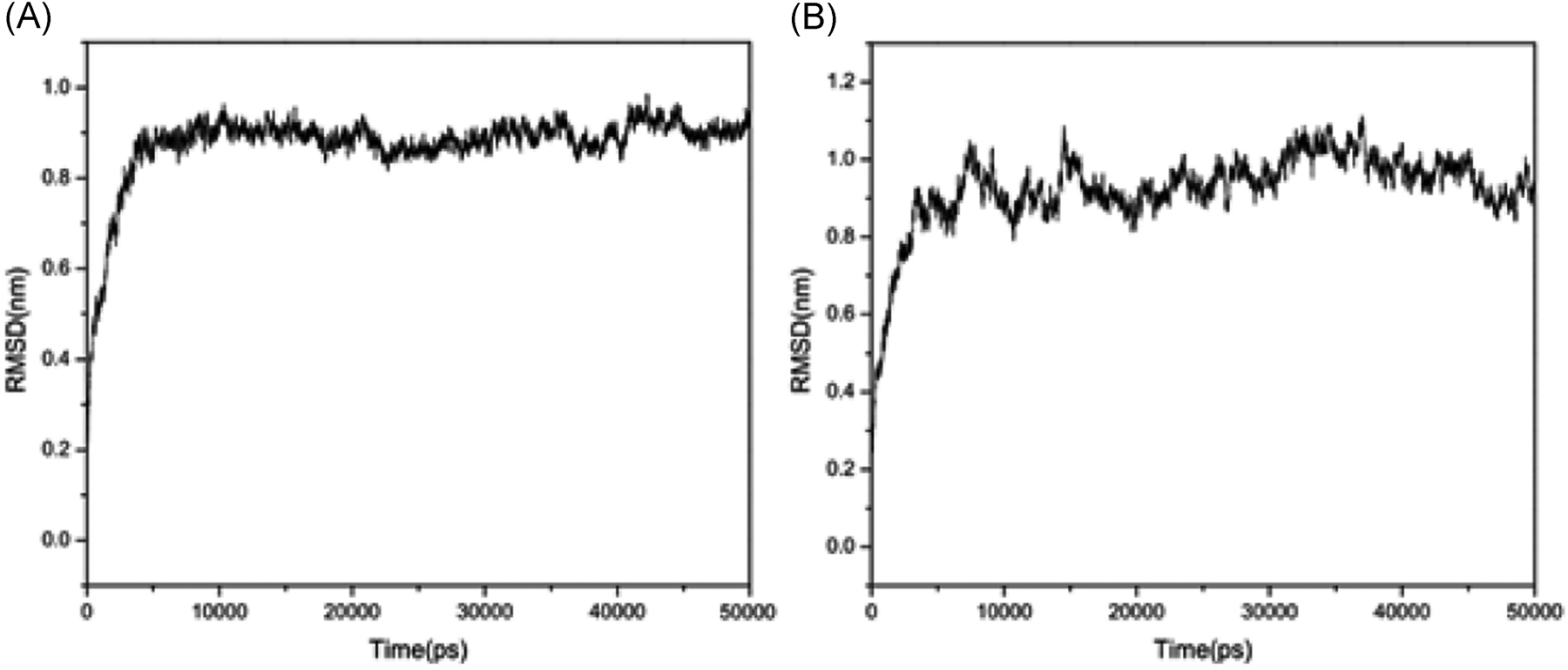
Dynamic trace of compound HBSC11 to the La protein (A); Dynamic trace of compound 5a to the La protein (B)

**Figure 8:**
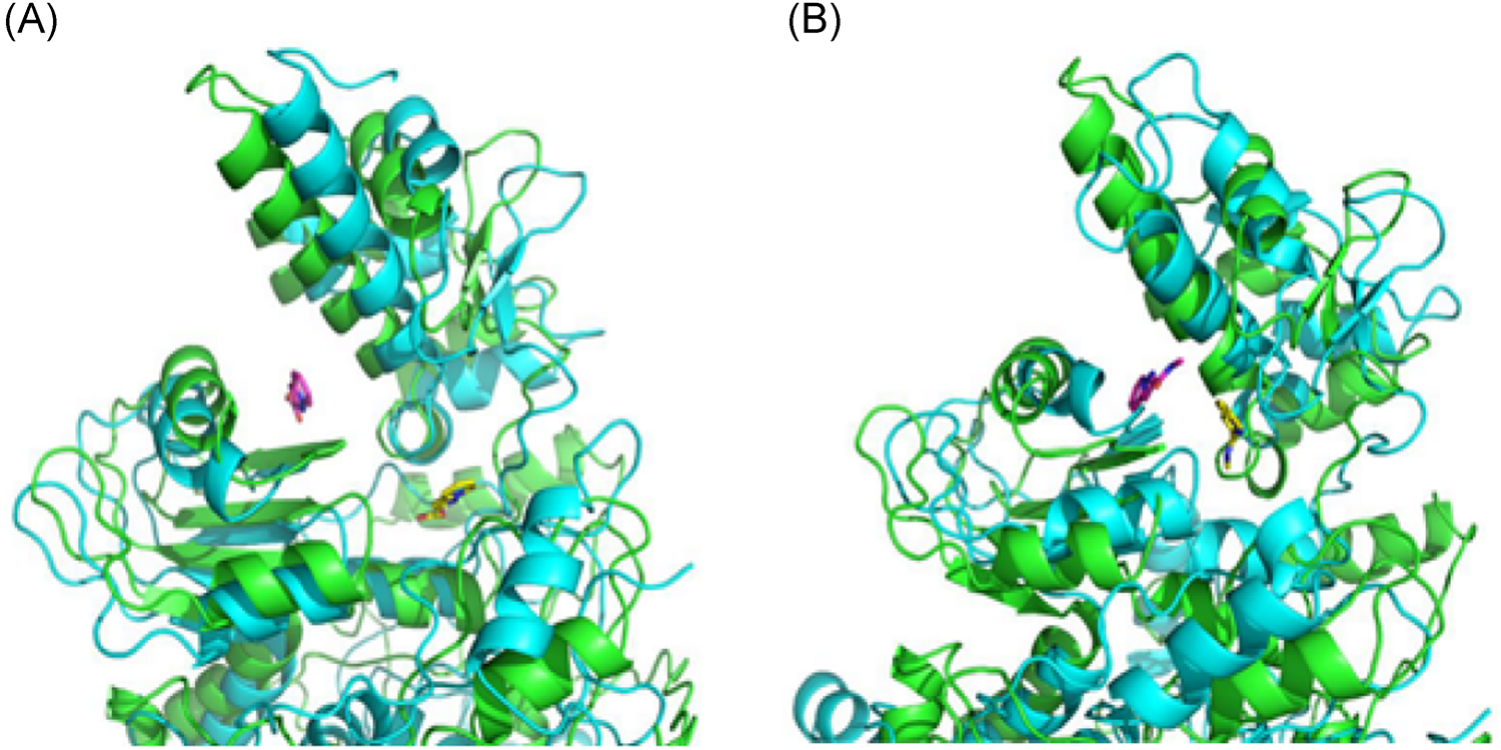
The initial and stable conformations of compound HBSC11 to the La protein (A); The initial and stable conformations of compound 5a to the La protein (B). The green cartoon is an initial conformation of La protein, the blue cartoon is the stable conformation of La protein. The rosy sticks are the initial conformation of compound, the yellow sticks are the stable conformation of compound

In the MD simulation studies, the score of leading compounds HBSC11 was −4.1443 and that of the candidate compound 5a was −4.1123, HBSC11 combined better than 5a with the La protein. At the end of 5 ns, the simulation dynamic trace of compound HBSC11 to the La protein runs into stationary phase (Figure 7A), whereas 5a needs 20 ns (Figure 7B). The initial and stable conformations of HBSC11 and 5a to the La protein are shown in Figure 8.

### 3.5 Prediction of physicochemical properties

As shown in Table 3, some physicochemical properties of HBSC11, 5a, 5b, and 3a were calculated using free online software. The results showed good compliance with the Lipinski's rule of five, which suggests their potential use as a drug.

**Table 3:** Prediction of physicochemical properties* of HBSC11, 5a, 5b and 3a

Compounds	nViol	MW	natoms	miLogP	nON	nOHNH	nrotb	TPSA	MV
range		<500		<5	<10	<5	<=10	<140	
HBSC11	0	176.18	13	1.57	4	0	2	43.61	153.78
5a	0	175.19	13	0.93	4	1	1	46.41	157.19
5b	0	217.27	16	2.05	4	1	3	46.41	207.38
3a	0	237.26	18	2.63	4	1	2	46.41	212.04

* n miLogP, molinspiration predicted LogP; MW, molecular weight; MV, molar volume; Viol, number of violations; natoms, number of atoms; nON, number of hydrogen bond acceptors; nOHNH, number of hydrogen bond donors; nrotb, number of rotatable bonds; TPSA, topological polar surface area.

## 4 DISCUSSION

La protein is a multifunctional nucleoprotein that is involved in diverse aspects of RNA metabolism and translation. Heise T and colleagues have focused on the relationship between La protein and HBV for many years; they found that La protein binds to the 5′ end of HBV RNA, which protects the HBV RNA against the nuclear RNase activity.^9^ Subsequently, other researchers used specific siRNA or La mutation to prove that La protein is involved in the life cycle of HBV RNA.^12^, ^22^, ^23^ These results indicate that La protein is a potential target for novel anti‐HBV strategies. In our previous study, we used virtual high‐throughput screening and biological evaluation and discovered a new La inhibitor with anti‐HBV activity.^13^ In this study, we aimed at optimization, synthesis, and evaluation of HBSC11 as a specific inhibitor of La protein.

Firstly, we identified and retained active sites between HBSC11 and La, and obtained 10 derivatives by introducing hydrophobic, hydrophilic and tricyclic groups containing aromatic rings on the side chain. Secondly, we screened three promising candidates with good anti‐HBV activity in HepG2.2.15 cells (compounds 3a, 5a, and 5b). Subsequently, we tested candidate compounds and HBSC11 in Hep AD 38 cell line which has a higher expression of HBV viral load. The results showed that 5a and 5b have better antiviral activity. Especially, 5a was the best hit with lower levels of HBsAg and HBeAg compared with HBSC11 after several days of treatment. Lastly, on the basis of in vitro experiments, we selected HBSC11, 5a, and 5b for further investigation of the antiviral activity and toxicity in a mouse model established by hydrodynamic injection.

All results indicated that compound 5a is a promising anti‐HBV compound. It reduced 50% of HBV antigen, which is comparable to the in vitro results of entecavir. In the mouse model, 5a showed 98.9% inhibition rate for HBV DNA, 57.4% for HBsAg, and 46.4% for HBeAg level; the corresponding rates in the control group were 90.8, 3.8, and 9.8%.

To characterize the mechanism of antiviral effect, we predicted the structure‐activity relationship. Investigation showed that 3d, 5a, and 5b were hydrophobic groups. It was found that the chain‐like substituents were superior to the cyclic ones which had high molecular weight and larger space resistance. This group was better than the hydrophilic group (3e, 5c, 5d) and the aromatic ring group (3a, 3b, 3c, and 3f). The results indicate that small‐molecule liposoluble compound exhibit better anti‐HBV activity. In line with immunofluorescence examination of liver tissue sections, the treatment group did show a decrease in La protein, especially in the nucleus, as compared to naive sections. Besides, the predicted physicochemical properties of 5a showed good compliance with Lipinski's rule of five. Moreover, the results of MD simulation studies showed that HBSC11 and 5a combine with La protein well. Thus, it seems worthwhile to continue our work to explore new anti‐HBV drugs.

Our study provides novel experimental evidence of the restraining effect of HBSC11 and its derivatives on HBV via their influence on La protein. However, there are several questions that are yet to be answered; it cannot be ignored that these compounds may have other potential targets, which implies that multifunction or side effects may occur. Recently, we used microarray and quantitative real‐time PCR to investigate the mechanism of H11‐mediated inhibition of HBV infection. The results showed that HBSC11‐induced inhibition of HBV replication was mediated via miR‐ 3912‐5p, miR‐6793‐5p, and miR‐7159‐5p and participation in the Wnt, beta‐catenin, and PPAR signaling pathways.^24^, ^25^ Further research is underway on this subject.

## CONFLICTS OF INTEREST

The authors declare that there is no conflict of interest.

## Supporting information

Supporting Information
