# Supplementary material for: Study on the structure optimization and anti‐hepatitis B virus activity of novel human La protein inhibitor HBSC11
Source: J Med Virol. 2019 Jul 12;91(10):1818–29. doi: 10.1002/jmv.25528 (PMC6771476; doi:10.1002/jmv.25528)

**Supplementary Material**

**Study on the structure optimization and anti-hepatitis B virus activity of novel human La protein inhibitor HBSC11**

**Table of contents**

1. **Representative HNMR and HPLC of 10 compounds S1**

**S1. Representative HNMR, HPLC and LC/MS of 10 compounds.**

**N-phenylpyrazolo[1,5-a]pyridine-2-carboxamide (3a)**: 309 mg, light yellow solid, yield 52.1%. 1H NMR (500 MHz, DMSO-d6) δ 10.39 (s, 1H), δ 8.75 (d, J=7.5, 1H), δ 7.89-7.82 (m, 3H), δ 7.38-7.32 (m, 3H), δ 7.14-7.07 (m, 3H).


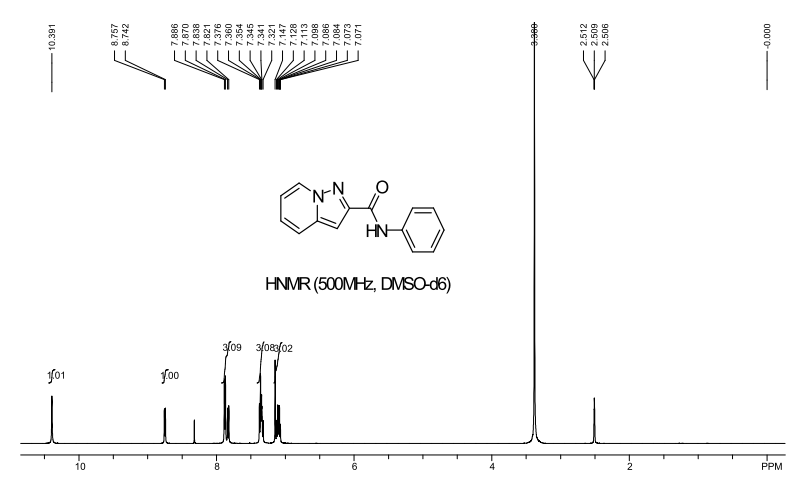


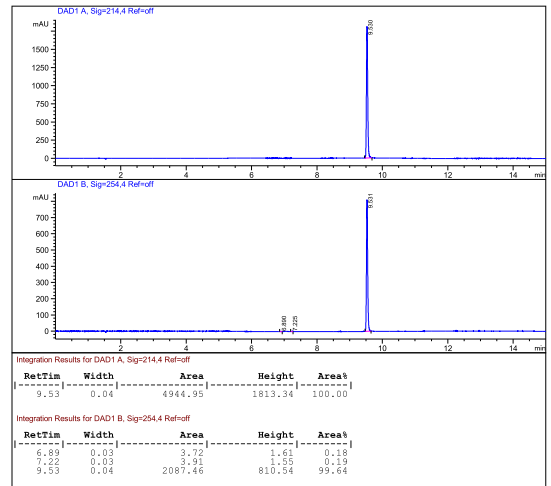


**N-benzylpyrazolo[1,5-a]pyridine-2-carboxamide (3b)**: 490 mg, white solid, yield 78.1%. 1H NMR (500 MHz, DMSO-d6) δ 9.05-9.02 (m, 1H), δ 8.69-8.68 (m, 1H), δ 7.78 (d, J=9.0, 1H), δ 7.35-7.22 (m, 6H), δ 7.05-7.02 (m, 2H), δ 4.49 (d, J=6.5, 2H).


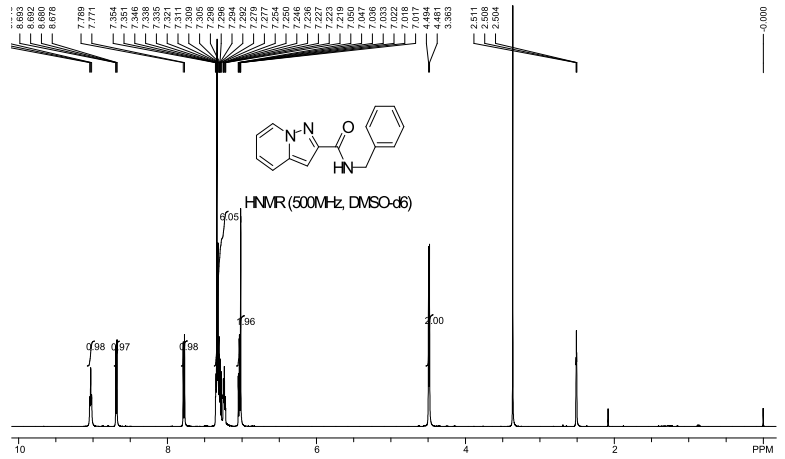


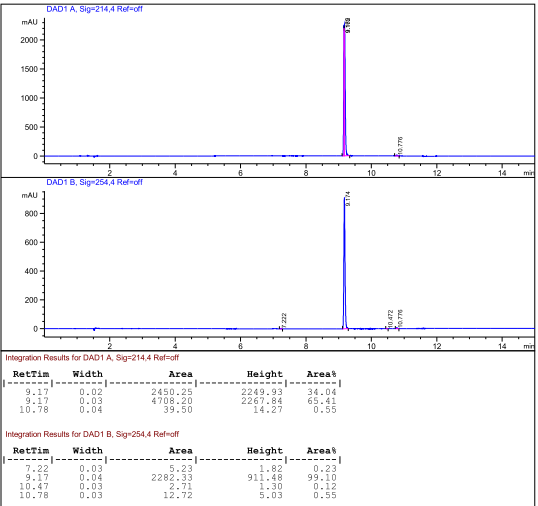


**N-(pyridin-3-yl)pyrazolo[1,5-a]pyridine-2-carboxamide (3c)**:

337 mg, light yellow solid, yield 56.6%. 1H NMR (500 MHz, DMSO-d6) δ 10.69 (s, 1H), δ 9.05 (d, J=2.0, 1H), δ 8.74 (d, J=9.0, 1H), δ 8.32-8.27 (m, 2H), δ 7.83 (d, J=10.5, 1H), δ 7.41-7.32 (m, 2H), δ 7.16-7.07 (m, 2H).


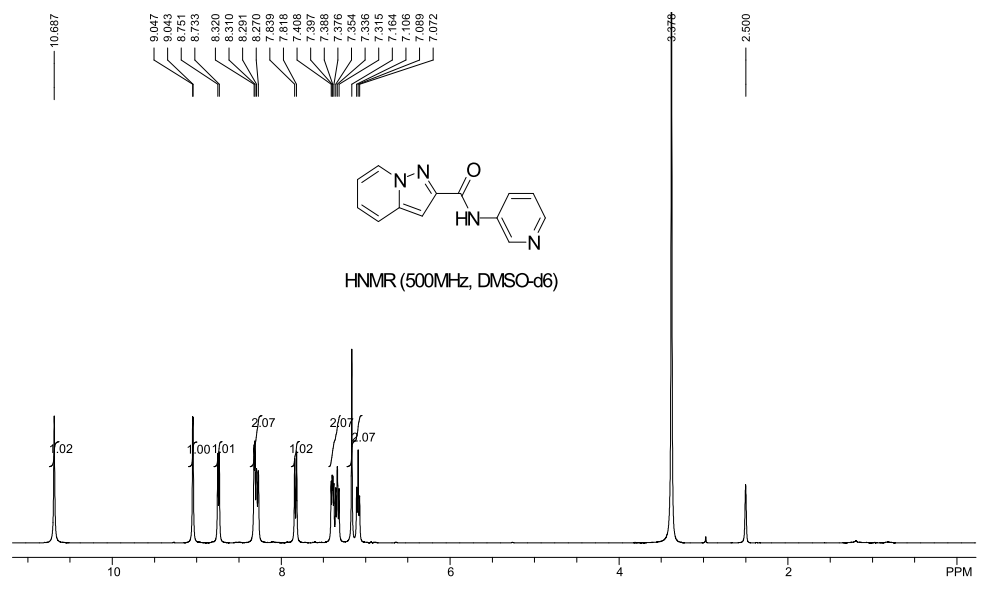


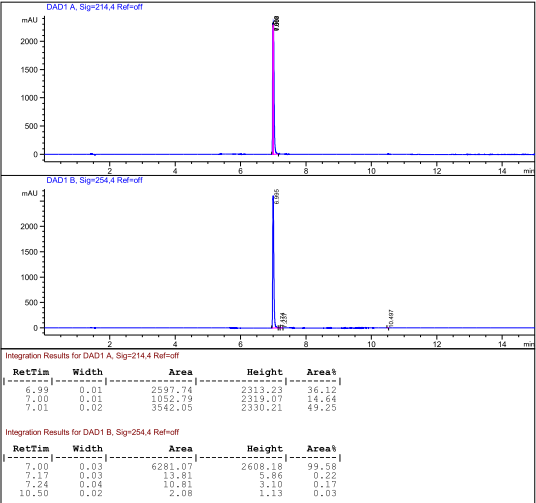


**N-cyclohexylpyrazolo[1,5-a]pyridine-2-carboxamide (3d)**: 373 mg, white solid, yield 61.4%.1H NMR (500 MHz, CDCl3) δ 8.38 (dd, J=9.0,1.5, 1H), δ 7.57 (d, J=11, 1H), δ 7.15-7.11 (m, 1H), δ 7.05-7.00 (m, 2H), δ 6.84 (td, J=9.0,2.0, 1H), δ 4.03-3.98 (m, 1H), δ 2.06-2.03 (m, 2H), δ 1.78-1.75 (m, 2H), δ 1.68-1.65 (m, 1H), δ 1.48-1.29 (m, 5H).


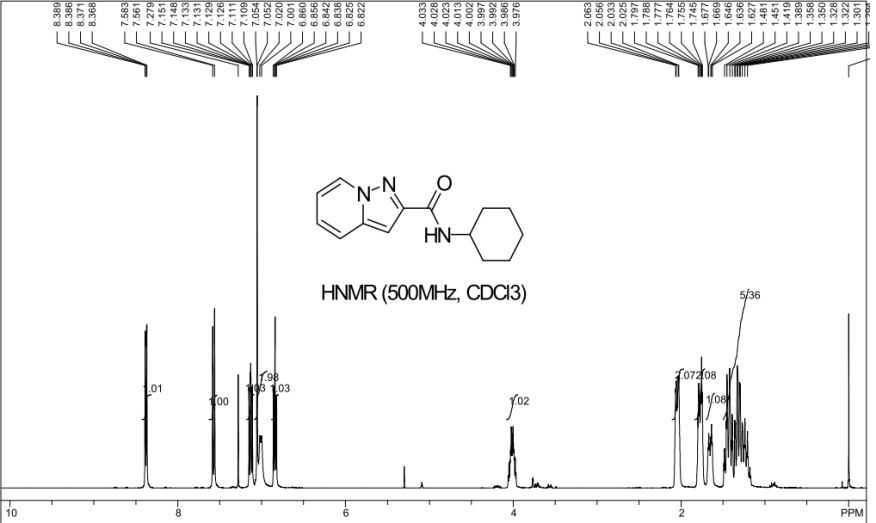


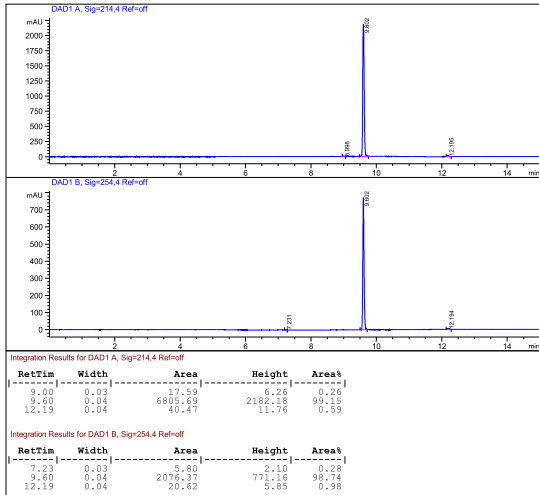


**Morpholino(pyrazolo[1,5-a]pyridin-2-yl)methanone (3e)**: 310 mg, white solid, yield 53.6%. 1H NMR (500 MHz, DMSO-d6) δ 8.71 (d, J=7.5, 1H), δ 7.75 (d, J=9.0, 1H), δ 7.29 (dd, J=16.5,7.0, 1H), δ 7.01-6.98 (m, 1H), δ 6.88 (s, 1H) , δ 3.87 (s, 2H) , δ 3.67 (s, 4H) , δ 3.60 (s, 2H).


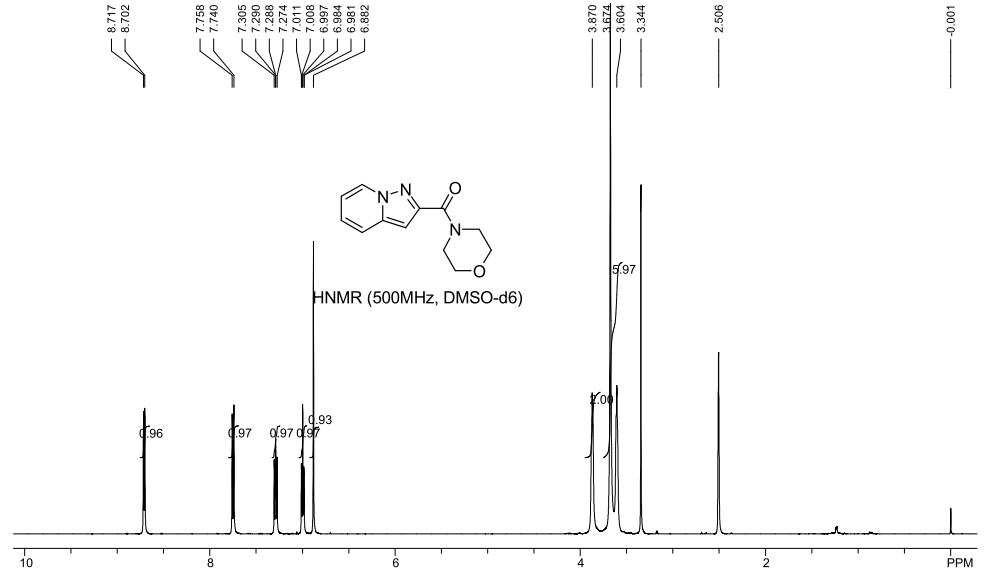


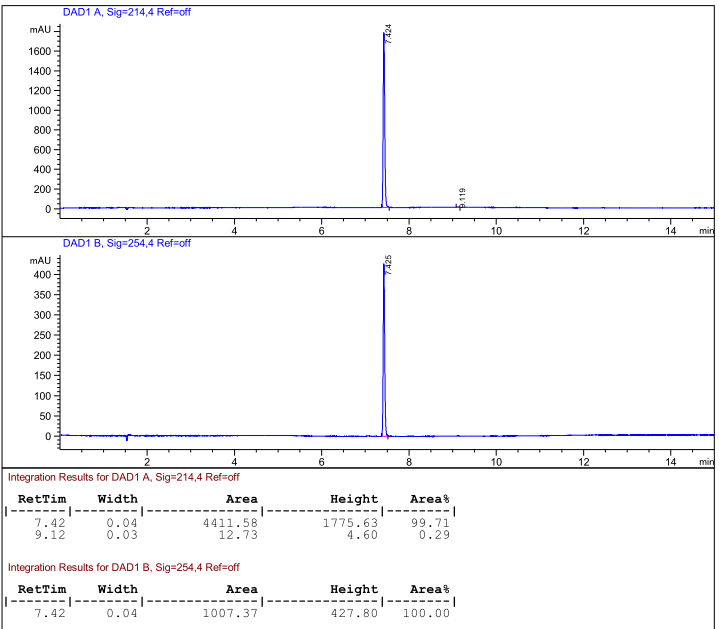


# N-(pyridine-4-yl)pyrazolo[1,5-a]pyridine-2-carboxamide (3f): 1H NMR (500 MHz, DMSO-d6) δ 10.85 (s, 1H), δ 8.76 (dd, J=8.8, 1.5, 1H), δ 8.49 (d, J=7.5, 2H), δ 7.90-7.92 (m, 2H), δ 7.50 (d, J=11, 1H) , δ 7.34-7.38 (m, 1H) , δ 7.20 (s, 1H) , δ 7.10-7.13 (m, 1H) , δ 1.34 (s, 2H)


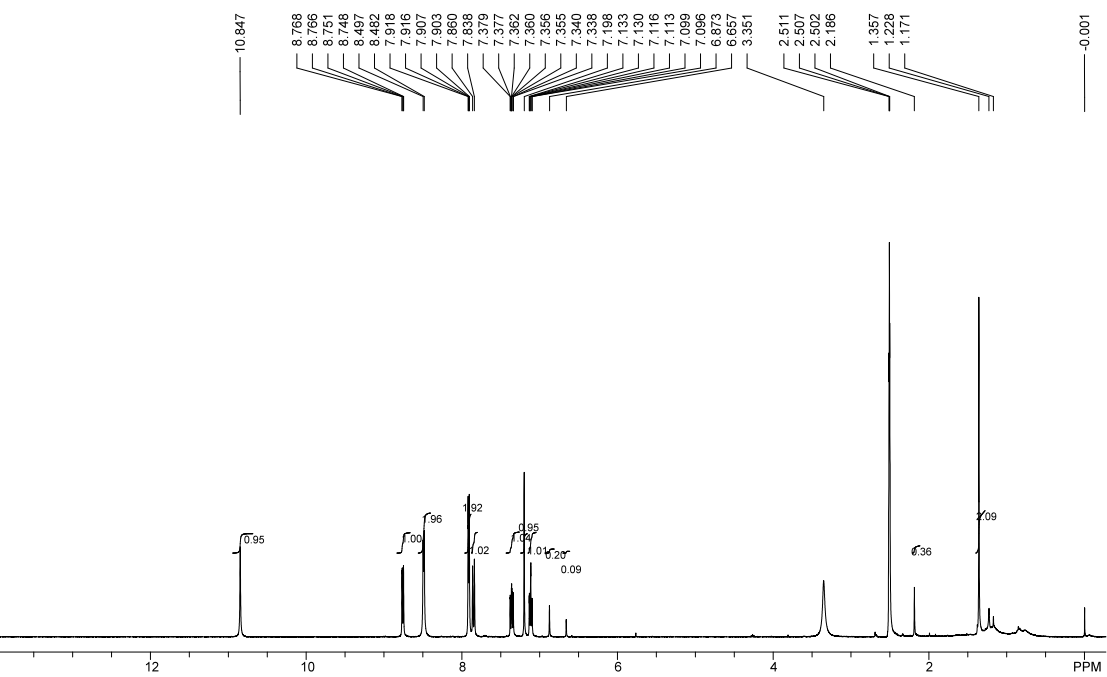


**N-methylpyrazolo[1,5-a]pyridine-2-carboxamide (5a)**: 310 mg, white solid, yield 50.6%.1H NMR (400 MHz, CDCl3) δ 8.37 (d, J=7.2, 1H), δ 7.59 (d, J=8.8, 1H), δ 7.14 (dd, J=8.8, 6.4, 2H), δ 7.11 (br. s, 1H), δ 6.85 (td, J=7.2, 0.8, 1H), δ 3.04 (d, J=7.2, 1H).


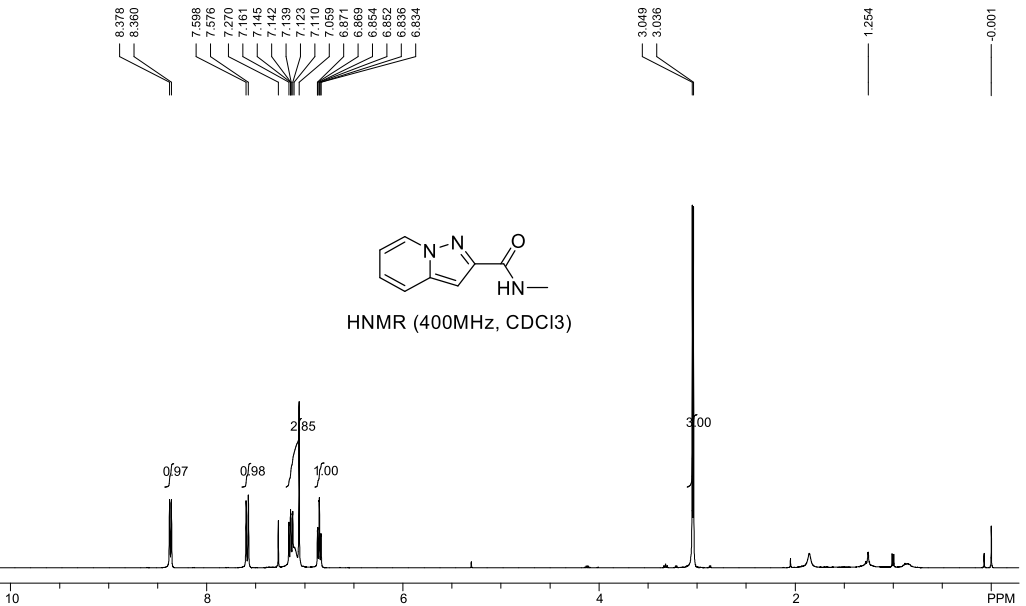


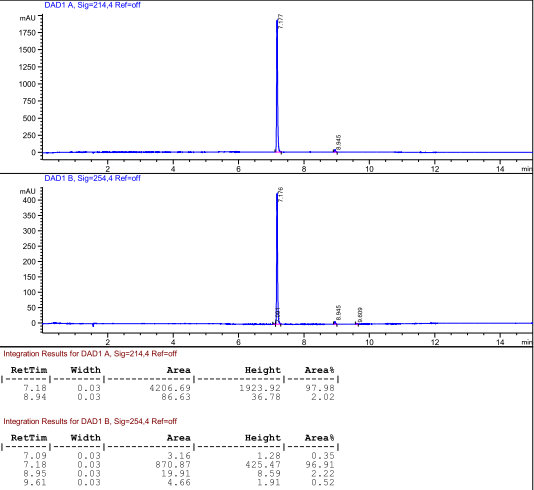


**N-isobutylpyrazolo[1,5-a]pyridine-2-carboxamide (5b)**: 323 mg, white solid, yield 42.5%.1H NMR (500 MHz, CDCl3) δ 8.39 (d, J=9.0, 1H), δ 7.59 (d, J=10.5, 1H), δ 7.18-7.12 (m, 2H), δ7.06 (s, 1H), , δ 6.87-6.84 (m, 1H), δ 3.32 (t, J=8.0, 2H), δ 1.98-1.88 (m, 1H), δ 1.00 (d, J=8.5, 6H).


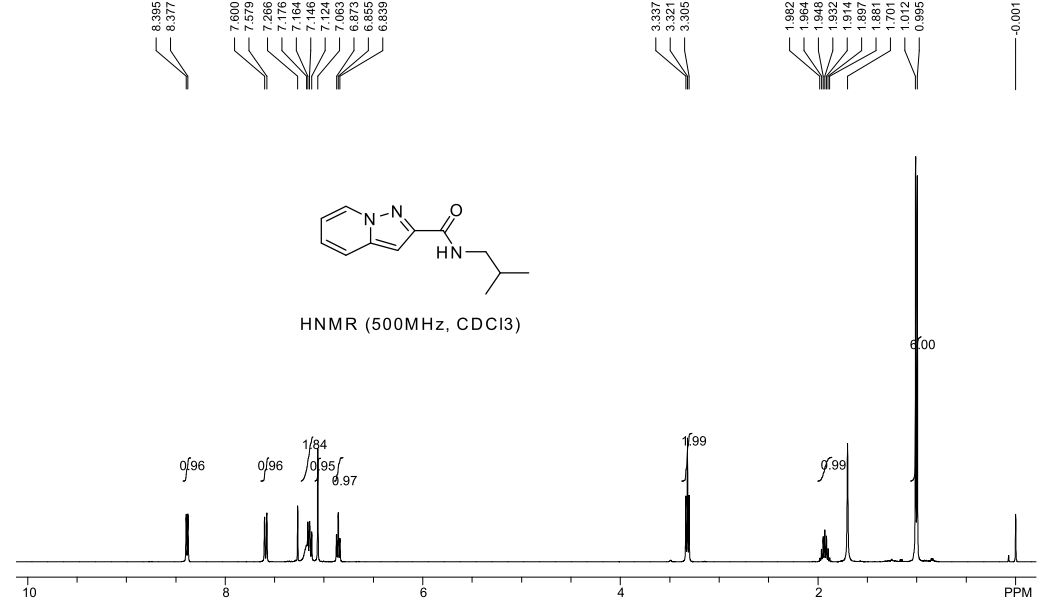


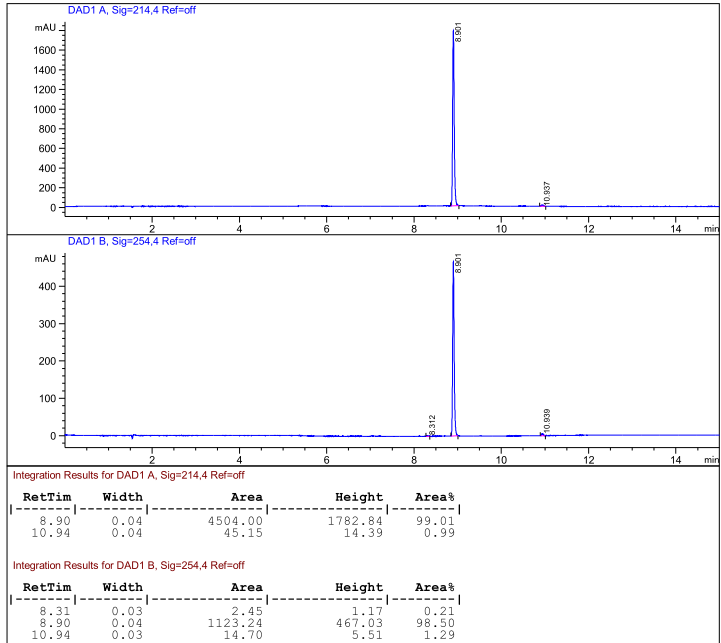


**N-(2-hydroxyethyl)pyrazolo[1,5-a]pyridine-2-carboxamide (5c)**: 251 mg, white solid, yield 35%.1H NMR (500 MHz, CDCl3) δ 8.36 (d, J=6.5, 1H), δ 7.57 (d, J=9.0, 1H), δ 7.16-7.12 (m, 1H), δ 7.04 (s, 1H), δ 6.87-6.84 (m, 1H) δ 3.87 (t, J=5.0, 2H), δ 3.68-3.65 (m, J=10, 2H), δ 3.16-3.14 (br. m, 1H).


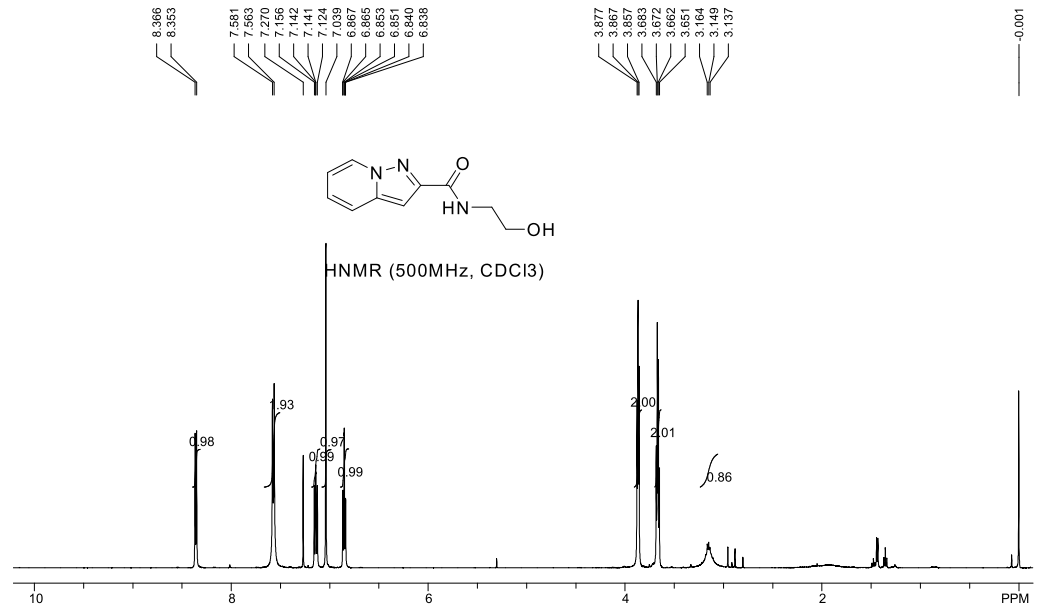


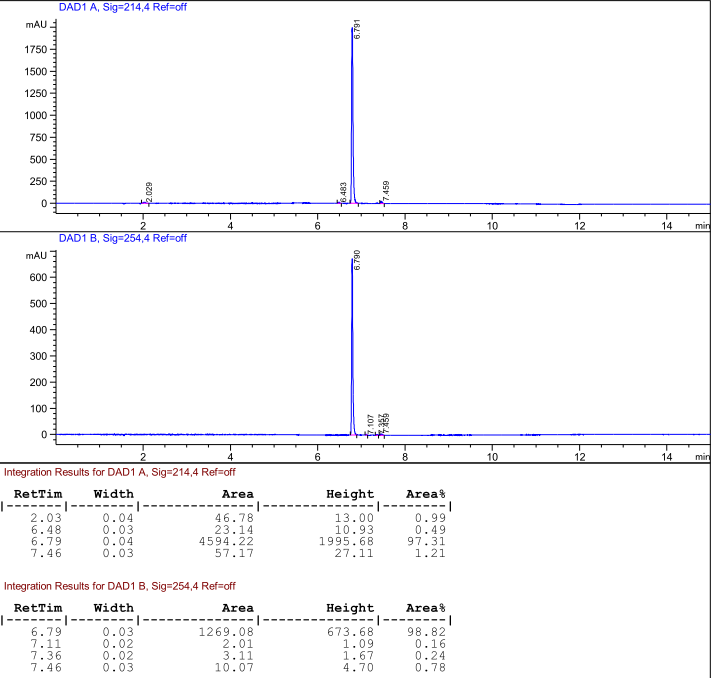


**N-(2-aminoethyl)pyrazolo[1,5-a]pyridine-2-carboxamide(5d)**: 65 mg, white solid yield 9.1%. 1H NMR (400 MHz, CD3OD) δ 8.54 (d, J=7.2, 1H), δ 7.67 (d, J=8.4, 1H), δ 7.26-7.22 (m, 1H), δ 7.01-6.96 (m, 2H), δ 3.74 (t, J=5.6, 2H), δ 3.24 (t, J=5.6, 2H).


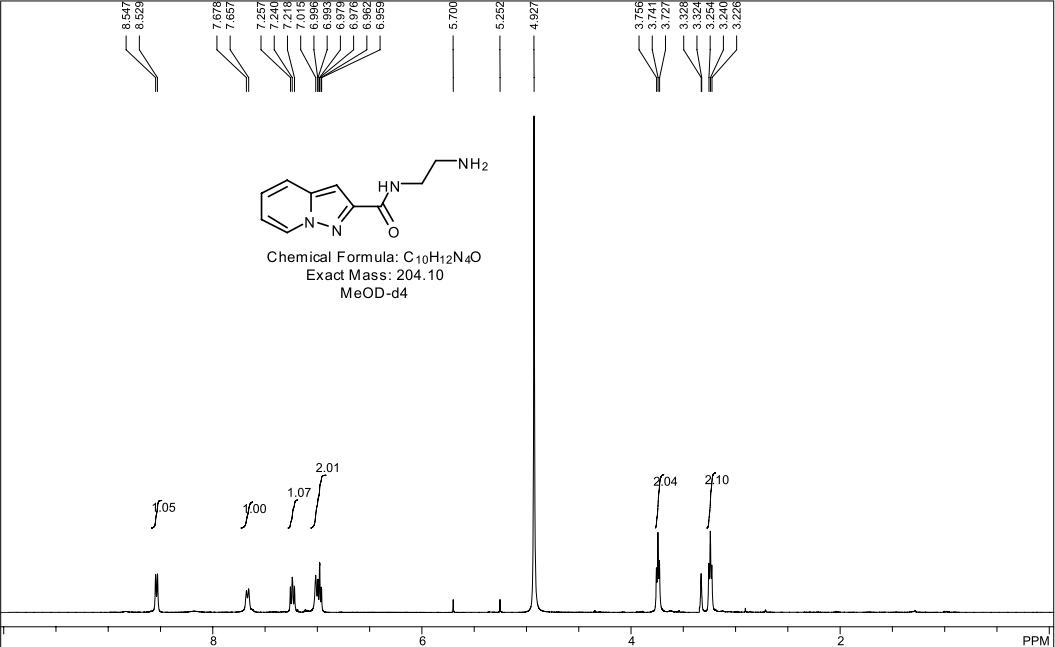


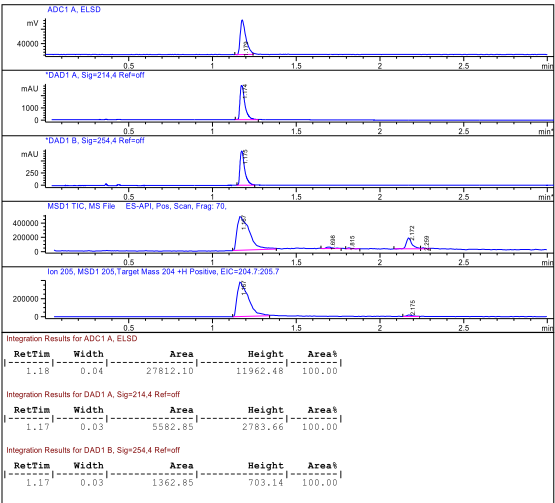

Supplement: Supplementary file 1 — Supporting Information [file JMV-91-1818-s001.docx]
